# Sequencing error correction without a reference genome

**DOI:** 10.1186/1471-2105-14-367

**Published:** 2013-12-18

**Authors:** Julie A Sleep, Andreas W Schreiber, Ute Baumann

**Affiliations:** 1 Australian Centre for Plant Functional Genomics, The University of Adelaide, Urrbrae, SA 5064, Australia; 2 Phenomics and Bioinformatics Research Centre, University of South Australia, Mawson Lakes, SA 5095, Australia; 3 ACRF South Australian Cancer Genome Facility, Centre for Cancer Biology, SA Pathology, Adelaide, SA 5000, Australia; 4 School of Molecular and Biomedical Science, University of Adelaide, Adelaide, SA 5000, Australia

## Abstract

**Background:**

Next (second) generation sequencing is an increasingly important tool for many areas of molecular biology, however, care must be taken when interpreting its output. Even a low error rate can cause a large number of errors due to the high number of nucleotides being sequenced. Identifying sequencing errors from true biological variants is a challenging task. For organisms without a reference genome this difficulty is even more challenging.

**Results:**

We have developed a method for the correction of sequencing errors in data from the Illumina Solexa sequencing platforms. It does not require a reference genome and is of relevance for microRNA studies, unsequenced genomes, variant detection in ultra-deep sequencing and even for RNA-Seq studies of organisms with sequenced genomes where RNA editing is being considered.

**Conclusions:**

The derived error model is novel in that it allows different error probabilities for each position along the read, in conjunction with different error rates depending on the particular nucleotides involved in the substitution, and does not force these effects to behave in a multiplicative manner. The model provides error rates which capture the complex effects and interactions of the three main known causes of sequencing error associated with the Illumina platforms.

## Background

The combination of a high read depth and the highly expressed nature of some sequences can result in some reads occurring millions of times in a next generation sequencing data set. For these situations, even very low error rates may still result in the presence of a multitude of sequence variants. Distinguishing these variants from true biological variants is a technological and computational challenge. In many species, this difficulty is compounded by the lack of an available reference genome.

The importance of identifying and correcting sequence errors has been highlighted by the recent discussion prompted by the report of the presence of widespread differences between the human genome (DNA) and reads derived from the corresponding RNA [1]. While it is tempting to interpret such differences as being due to the presence of RNA editing, a reanalysis of this same data set showed that the majority of the reported differences were actually consistent with technical artefacts arising from sequencing errors (see, e.g. [2]).

It goes without saying that when the genome of an organism has not been sequenced and assembled, the difficulty of identifying possible sequencing errors is greatly increased, necessitating the development of alternate analysis methods.

Sequencing errors arising from the use of Illumina sequencers, on which we concentrate, can occur for a variety of reasons. One source of error originates from a phenomena referred to as crosstalk. Crosstalk occurs when there is an overlap in signals of the dye emission frequencies used in sequencing machines.

This overlap can lead to confusion of the nucleotide G with nucleotide T, and of A with C [3,4]. A second cause of error is referred to as either *dephasing* or *phasing*. Since sequencing is done in cycles, an error in an earlier cycle may propagate to and affect later cycles. This usually results in the errors appearing more frequently toward the ends of the reads. T fluorophore accumulation is another source of error, and results in more T’s being incorrectly attributed towards the ends of reads. For an extensive review, see [5], which also discusses other possible sources of sequencing errors such as signal decay, mixed clusters and boundary effects. Additionally, sequence-specific error patterns, including inverted repeats and the effects of the nucleotide sequence GGC have been proposed as an important cause of sequencing errors through dephasing [6].

The issue of sequencing errors is so ubiquitous that being able to detect and correct them is essential in many areas of molecular biology, particularly in the identification of miRNAs. In [7], the occurrence of errors and their corresponding rates were investigated by looking at Illumina data sets (2.8 million 27-base reads) taken from *Beta vulgaris* and *Helicobacter acinonychis*. By aligning reads to the known genomes of these bacteria, error rates were derived for each of the 12 possible nucleotide substitutions.

This work is typical of procedures that rely on the availability of a reference genome and many methods and software packages have been developed for the detection and/or correction of sequencing errors in this setting [8]. One such method [9] is based on an algorithm for correcting sequencing errors that uses a ‘generalized suffix trie’. However, this method requires a reference genome and assumes a uniformly distributed error rate. A similar method using suffix arrays is that of Ilie et al. [10]. An alternative method for correcting short reads that takes into account genomic repeats, is described in [11]. Based on a position-dependent error model, error probabilities are estimated for each nucleotide substitution type. The method of [12] also requires a reference genome, has a position dependent error model, but it is one that is not base-specific.

A different approach, that does not rely on the existence of a sequenced genome, was adopted in [13]. Short reads are clustered into trees where the most abundant sequence is taken to be the root of a tree, and “children”, who differ by *n* nucleotide substitutions, are placed at the *n*th level. These children are classified either as sequencing errors or biological variants. This approach utilises the Illumina quality scores, which are adjusted by means of actual error rates determined by BAC sequencing data used as a control. These error rates are used to estimate the expected number of errors for a given position *pos*, quality value *Q*, and substitution pattern *R* (e.g. A → C) by calculating

(1) $$N_{\text{error}}(\text{pos},Q,R)=\frac{P_{\text{error}}}{1-P_{\text{error}}(\text{pos})}N_{\text{correct}}[\;\text{Rate}(Q,R)]$$

where *P*_*error*_ is the overall probability of an error, *P*_*error *_(*pos*) is the adjusted probability of an error at each position, and *Rate *(*Q*,*R*) gives the probability of pattern *R* occurring when the quality score takes the value *Q*. For each child, the expected number of errors are compared to the actual frequency, using a *Z*-test with the null hypothesis being that the sequence read contains a sequencing error.

**Figure 1:**
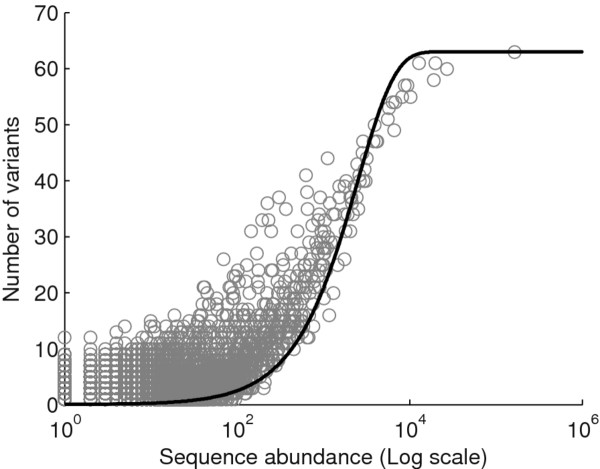
**Number of vertices plotted against sequence abundance.** Number of vertices for each parent node (Y) plotted against abundance (X) for sequences of length 21. The theoretical curve given by the function *Y* = 3*L *[ 1 - (1 - *p*)^*X*^] ([14]), using *p* = 0.0004 is shown in grey. This function explains the general trend of the data but not the substantial variation in number of variants.

A probabilistic model for predicting the occurrence of sequencing errors in short RNA reads proposed in [14] does not rely on the availability of a sequenced genome nor on platform-provided quality scores. Instead, it is based on the observed frequencies of the sequence variants. A graph is constructed where reads are connected if they differ by a single nucleotide substitution. Examples of a graph of this type can be seen in Additional files 1 and 2. Next, the number of single nucleotide variants for each sequence is plotted against the abundance of the sequence (see e.g. Figure 1). The appropriateness and advantage of using graphs of connected single nucleotide variants becomes apparent by studying the close relationship between sequence abundance and the number of vertices emanating from the corresponding node in the graph. A probability, *p*, was obtained by fitting the data with the curve describing the expected number of single nucleotide variants for a sequence of given abundance *X*

(2) $$Y=3L[\;1-{\left(1-p\right)}^{X}]$$

where *L* is the length of the sequence. Children of a given sequence are then classified as true biological variants if their abundance is > > *pX*, where *X* is the abundance of the ‘parent’ sequence. If their abundance is roughly *pX* or less, they are considered to be sequencing errors. Additionally, reads with an abundance of less than 12 are also removed. This approach has the advantages of not requiring a reference genome or platform-provided quality scores. However, a limitation of this model lies in the assumption of a constant error rate along the read. While the number of sequence variants does increase with increasing abundance of a sequence, there is considerable variation (as seen in Figure 1) that cannot be explained by a probabilistic model based on a constant error rate.

In the following sections we present a method for modelling sequencing errors, extending the graph-based approach described in [14] but incorporating position- and substitution-specific error rates. Additionally, we do not enforce these effects to be working multiplicatively, and the method does not require a reference genome or quality scores. Results are included that highlight the advantages of the method in its ability to account for the interactions between the different causes of sequencing errors.

## Methods

We extend the approach of [14] by allowing for error rates that are both dependent on position along the read and vary by each nucleotide substitution pattern (e.g., T → G). As stated earlier, we do not restrict ourselves to the assumption that these two effects work in a multiplicative fashion. The method was tested on data sets obtained from high-throughput sequencing of short RNA reads extracted from the leaves and roots of three different cultivars of wheat. For this purpose, the Illumina Solexa sequencing technology was employed.

The number of reads for each sample and lane ranged from 5 to 41 million reads, and several sequencing runs were performed approximately two years apart. The first samples were 36 base reads run on the Illumina Genome Analyzer (GeneWorks Pty. Ltd., c. 2009) and the second were 50 base reads from Illumina HiSeq with Illumina TruSeq v3 reagents (Australian Genome Research Facility Ltd., December 2011). What we term an individual data set is the sequenced data from a particular lane (numbered between 1 and 8), corresponding to their physical locations on the flow cell. We refer to lanes 4 and 5 as being the innermost lanes and 1 and 8 as the outermost.

### Data processing and graph construction

Processing began with the 3^′^ adaptors being trimmed from the sequences. A number of mismatches to this adaptor were allowed depending on the length of the matching sections, as described in [14]. Reads containing homopolymer tracts were not removed at this early stage. Removal occurred as a final step after the error model is built and the data corrected for sequencing errors. This was done so as to prevent the removal of any parent sequences of erroneous reads. Reads containing undetermined nucleotides (denoted by the letter N) were, however, excluded from our analysis. Sequences of length outside the region of interest (20-24 nucleotides) were not studied any further. Unique sequences were identified along with the frequency (abundance) with which each was seen in the data.

Graphs were constructed, according to the model of [14], by joining sequences that are single nucleotide variants for each length of sequence. These graphs are then decomposed to find the disconnected subgraphs. Table 1 contains details of the size and number of these disconnect subgraphs for one of the example data sets. An example of two of these disconnected subgraphs are shown in Additional file 1 and Additional file 2. Subgraphs such as these, were analysed further to develop a model of the error rates in their respective data sets and used to identify sequencing errors.

**Table 1:** Properties of created subgraphs

**Illumina GA lane 2**					
**Length**	**Frequency of subgraph sizes**				**Largest**
	1	2-20	20-40	40 +	
20	33,170	2,530	27	17	992
21	132,373	11,992	170	105	1,048
22	86,118	7,078	63	32	387
23	171,287	9,714	79	47	296
24	1,277,008	101,108	1,264	757	2,030

Excluding adaptor trimming, the graphs are created in approximately 40 minutes (on a single processor of a PC running 32 bit Windows XP with 3.45GB of RAM) for a file of 6 million 35-base reads. Our algorithm was not parallelised, but can be, which would greatly reduce the processing time. A similar amount of time is required for the building of error models and correction of the graphs. The full source code is publicly available [15].

### Error model

More reliable error statistics can be extracted from sequences that appear a large number of times and have many sequence variants. Hence, for this purpose, we have chosen to select a subset of large subgraphs based on a user-defined threshold on the minimum number of nodes. These large subgraphs are then used to build a model of the error rate. Furthermore, to exclude as many graphs containing a true biological variant as possible, we introduce an additional series of thresholds, *t*, for how much of the total abundance is attributable to the parent node

$$\frac{a_{\text{parent}}}{a_{\text{total}}}\geq \frac{t}{100}\;t\in \{30,70,75,80,85,90\}$$

where *a*_*parent*_ is the number of times the parent sequence appears in the data set and *a*_*total*_ is the sum of the frequencies of sequences in the subgraph. Starting from graphs satisfying the highest parental abundance threshold, we analyse the children of the most abundant sequence, recording the abundances of the sequence and each child sequence, the position along the read where the child differs from the parent, and the nucleotide substitution that has occurred.

From this information we calculate, for each graph, a probability of error for each combination of nucleotide substitution pattern type and position along the read. We use a weighted average (weighted on the basis of the abundance of the parent sequence) of all the individual probabilities, to determine our overall probability estimates. For example, given estimates $\hat{P_{k}}({\text{pos}}_{i},R_{j})$, *k* = 1,…,*K*, for a position *pos *_*i *_and substitution pattern *R*_*j*_, we would calculate our probability estimate using the following formula

$${\hat{P}}_{\text{error}}(\;{\text{pos}}_{i},R_{j})=\frac{\underset{k}{\sum }(a_{{\text{parent}}_{k}}\times {\hat{P}}_{k}(\;{\text{pos}}_{i},R_{j}))}{\underset{k}{\sum }a_{{\text{parent}}_{k}}}$$

where $a_{{\text{parent}}_{k}}$ is the parental abundance of the parent sequence used to estimate $\hat{P_{k}}(\;{\text{pos}}_{i},R_{j})$.

Using this estimate, and assuming that our data may be modelled as coming from a binomial distribution *B *(*n*,*p*), we calculate 95% confidence intervals. The parameters used in the binomial distribution are *n*, being the number of sequences, given by $a_{{\text{parent}}_{k}}$ and *p*, being the error rate ${\hat{P}}_{\text{error}}({\text{pos}}_{i},R_{j})$. Those estimates $\hat{P_{k}}({\text{pos}}_{i},R_{j})$ lying above this confidence interval, and thus most likely to be derived from biological variants, are precluded from being used in the error model, and the weighted average and confidence interval is then recalculated. To ensure that positions with no sequencing errors and only biological variants (which would not be removed by the confidence interval method described above) did not contribute exceptionally high data points, an additional smoothing technique was employed. This involved adjusting probability estimates that were greater than twice the average of their two nearest neighbours. These unusually high values were replaced with this average.

**Figure 2:**
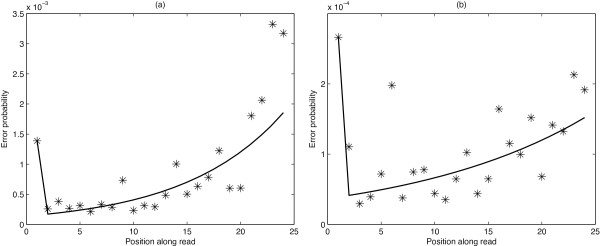
**Example model fit.** Data points and fitted model for the probability of an A being misread as a C, for **(a)** an Illumina GA data set and **(b)** an Illumina HiSeq data set.

We perform these calculations, as described in the preceding paragraph, beginning at parental abundance ratio 90% and working downwards. While the higher thresholds provide more reliable estimates, the number of graphs selected is not large and therefore all possible nucleotide substitutions are not seen at every position along the reads. Thus, we employ an iterative process to fill gaps in our estimates with probabilities derived from the subset of graphs with the next highest proportion threshold. Thereby, we have derived error probability estimates for all or most of the nucleotide transitions at each position along the read. We found that exponential curves provided a satisfactory fit to the data and provided the best theoretical fit to the expected error increase due to the phasing phenomenon. Consequently, we fitted exponential curves to these error estimates for each transition type between positions 2 and 24. This helped to further eliminate any effects of outliers (i.e., true biological variants) that were not rectified in the previous steps, and provided values for substitution-position combinations that were not observed in previous steps. An example of this, for Illumina GA data and the case of A being misread as C, is shown in Figure 2(a). Corresponding error rates for Illumina HiSeq data are an order of magnitude lower (Figure 2(b)). The error rate in the first position along the read is not fit to the exponential curve as, in the majority of cases, it was found to be much higher than the error rate in position 2. This is consistent with what was observed by [16], who attributed this to the lower intensity values that result from the longer handling time at the commencement of a sequencing run.

Our method does not assume that position and error type effects work multiplicatively. Our generalisation to account for these effects is simply

(3) $$\begin{array}{c} N_{\text{error}}(\text{pos},R)=\frac{P_{\text{error}}(\text{pos},R)}{1-P_{\text{error}}(\text{pos},R)}\times N_{\text{correct}}(\text{pos},r_{1}) \end{array}$$

where R is the nucleotide substitution pattern *r*_1_ → *r*_2_. Note that we do not enforce that

(4) $$P_{\text{error}}(\text{pos},R)=P_{\text{error}}(R)\times P_{\text{error}}(\text{pos})$$

and hence are able to model non-multiplicative effects.

The model described above is used to find and correct sequencing errors by comparing the observed sequence abundances with those predicted by the model. Statistical hypothesis testing is used for this purpose with the null hypothesis being that a given sequence is a sequencing error. Sequences for which the null hypothesis is rejected are classified as true biological variants, the remaining sequences are classified as sequencing errors.

## Results and discussion

**Figure 3:**
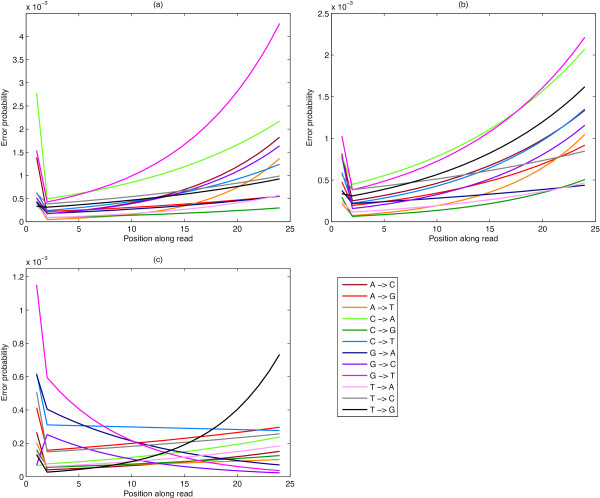
**Modelled error rates.** Modelled error rates from **(a)** an Illumina GA data set (lane 2), **(b)** an Illumina GA data set (lane 4) and **(c)** an Illumina HiSeq data set (lane 2).

Modelled error rate results for a selection of data sets are shown in Figure 3 and Table 1. For each figure the y-axis represents the probability of an error occurring as calculated by our model. Table 1 shows the model parameter values corresponding to Figure 3, for each of the nucleotide transitions for position 1, and for the parameters of the fitted exponential curve, *Ae*^*bx*^.

### Illumina GA

The G → T substitution error rate, which is the highest in the Illumina GA data sets (Figures 3(a) and (b)) can be attributed to the combined effects of cross-talk and T fluorophore accumulation. The transition C → A is also high, which can also be attributed to cross-talk. From Table 2 we can see, however, that the A → T error rate is the one that is increasing at the greatest rate at the ends of the reads, indicated by the largest exponent *b*, and this can be attributed to T fluorophore accumulation. The overall error rates found for these data concur for the most part with those reported in [7]. However, our model demonstrates that the error rate effects of both position and nucleotide transition type, do not work multiplicatively. The main reason for this lack of factorisation appears to be due to T fluorophore accumulation, which increases toward the ends of the reads. Thus, a non-multiplicative model, such as proposed here, is necessary to account for this phenomenon.

**Table 2:** Summary of modelled error probabilities and model parameters

	**Illumina GA lane 2**			**Illumina GA lane 4**			**Illumina HiSeq lane 2**		
**Error**	**Position 1**	***A***	***b***	**Position 1**	***A***	***b***	**Position 1**	***A***	***b***
A → C	1.4E-03	1.4E-04	0.11	8.2E-04	2.2E-04	0.08	2.7E-04	3.7E-05	0.06
A → G	5.1E-04	2.0E-04	0.04	4.8E-04	1.7E-04	0.07	4.1E-04	1.5E-04	0.03
A → T	4.1E-04	3.4E-05	**0.15**	2.1E-04	5.8E-05	**0.12**	2.0E-04	5.6E-05	0.03
C → A	**2.8E-03**	**4.3E-04**	0.07	7.9E-04	**3.9E-04**	0.07	6.6E-05	6.9E-05	0.05
C → G	4.2E-04	8.0E-05	0.05	2.9E-04	5.3E-05	0.09	1.6E-04	5.2E-05	0.04
C → T	6.3E-04	2.1E-04	0.07	5.9E-04	1.9E-04	0.08	6.2E-04	3.1E-04	-0.01
G → A	4.3E-04	1.6E-04	0.05	3.7E-04	2.0E-04	0.03	6.1E-04	4.7E-04	-0.08
G → C	5.1E-04	1.4E-04	0.10	7.8E-04	1.3E-04	0.09	6.9E-05	3.1E-04	-0.11
G → T	1.5E-03	3.5E-04	0.10	**1.0E-03**	3.3E-04	0.08	**1.2E-03**	**7.7E-04**	-0.13
T → A	3.6E-04	7.4E-05	0.08	2.4E-04	1.0E-04	0.06	1.4E-04	5.4E-05	0.05
T → C	6.1E-04	3.5E-04	0.04	5.6E-04	3.6E-04	0.04	5.1E-04	1.4E-04	0.02
T → G	3.3E-04	2.8E-04	0.05	3.4E-04	2.7E-04	0.08	1.3E-04	2.0E-05	**0.15**

Probabilities for position 1 and exponents of the fitted exponential curves, *Ae*^*bx*^, for positions 2 to 24 for the data sets corresponding to Figure 3.

By comparing Figure 3(a) with Figure 3(b), it can be seen that in GA datasets there is a strong dependence of overall error rate on the sequencing lane, with error rates lowest in the inside lanes (Figure 3(b)). Whether this is a more general phenomenon requires further investigation. However, it highlights the necessity of processing lanes separately.

### Illumina HiSeq

The error profiles of the sequenced reads from lane 2 of the Illumina HiSeq data (Figure 3(c)) show a qualitatively different profile in that some error rates are initially decreasing along the reads. Error rates along the read are substantially lower overall. The substitution G → T is higher at the beginning of the reads but becomes lower moving along the read, which is in contrast to the reverse error, T →G, which increases towards the end. The reason for this phenomena is unclear but it is hypothesised as due either to altered chemistry (the washing away of T fluorophores becoming more (too) effective) or to the changes in the different base calling algorithm (overcompensation for the T fluorophore accumulation phenomenon). We consider the latter scenario more likely as we see similar patterns for many of the other corresponding pairs of nucleotide substitutions. Moving along the first 24 bases of the read, C → T, G → A and G → C also decrease, while their reversed substitutions T → C, A → G and C → G increase. A lane trend is also observed in the Illumina HiSeq data. However, it does not involve all nucleotide transitions. The outer lanes, 2 and 8, have significantly higher error rates for the G → T and T → G transitions. These two error rates become progressively lower toward the inside lanes. The other nucleotide transition error rates remain essentially constant across the lanes.

### Evaluation

To address the difficult matter of evaluation we undertook two benchmarking analyses. Firstly, we applied our model to a simulated data set and secondly we checked the performance of our model by correcting reads from an organism with a known reference genome.

In our creation of a simulated data set, for the sake of comparison, we used the error probabilities from each position and transition that were found in the Illumina GA lane 2 data set. We then took a data set of short RNA reads thought to contain no sequencing errors and randomly simulated errors based on the given error rates and the corresponding binomial distributions. The data set was processed by our method in the same way as the other data sets. The resulting error model parameters are summarised in Table 3. A plot of this error model can also be found in Additional file 3. By comparing Table 3 to the first 3 columns of Table 2, it can be seen that the reconstruction of the error rates using the simulated data is very close. The same parameters are highest in both data sets, and all parameter values are of the same order of magnitude.

**Table 3:** Summary of model parameters resulting from simulated data

	**Simulated data**		
**Error**	**Position 1**	***A***	***b***
A → C	1.4E-03	1.8E-04	0.10
A → G	6.8E-04	2.3E-04	0.04
A → T	4.6E-04	4.6E-05	**0.15**
C → A	**3.0E-03**	**4.7E-04**	0.07
C → G	4.3E-04	8.0E-05	0.08
C → T	8.3E-04	2.4E-04	0.06
G → A	4.9E-04	2.0E-04	0.06
G → C	5.3E-04	1.4E-04	0.11
G → T	1.8E-03	2.7E-04	0.14
T → A	4.1E-04	1.2E-04	0.06
T → C	5.9E-04	2.9E-04	0.06
T → G	3.9E-04	4.5E-04	0.02

Probabilities for position 1 and exponents of the fitted exponential curves, *Ae*^*bx*^, for positions 2 to 24 for the simulated data set. The corresponding figure is shown in Additional file 3.

To further evaluate our model we studied HiSeq reads from a publicly available PhiX data set (SRA accession number SRS267273; SRX101468) [17]. After correcting the reads using our algorithm as described in the Methods section, we mapped our modelled correct and erroneous reads to a copy of the PhiX genome [18], obtaining a sensitivity measure of 99.29% and specificity of 96.64%. In the context of our error correction problem, this means that our algorithm retained 99.29% of correct sequencing reads, and identified 96.64% of the erroneous reads. The mapping was performed only up to 3 mismatches due to limitations of the mapping software. However, using further mismatches may result in an increased specificity measure. The results of the evaluation are shown in Table 4.

**Table 4:** Evaluation of error correction algorithm on PhiX genomic sequences

	**Model prediction**	
**Genome mapping**	**Correct sequences**	**Erroneous sequences**
Exact match	10115	8
1 mismatch	2779	64137
2 mismatches	164	17636
3 mismatches	14	3217

Sequence counts comparing our model predictions of correct and erroneous sequences to results obtained by mapping the sequences to the corresponding genome.

## Conclusions

We have proposed a model of sequencing errors that is flexible enough to incorporate known sources of error intrinsic to the Illumina sequencing technologies and that does not rely on the availability of a reference genome for error detection. We have demonstrated the advantages of using of a non-factorisable model, particularly necessitated by the presence of accumulated T fluorophores in the Illumina GA data, and other unknown non-multiplicative effects in the Illumina HiSeq data. The method described herein is potentially applicable not only to short RNA reads but also to other sequencing activities where a reliable sequenced genome is not available, such as in the field of metagenomics, where a mixed sample containing reads from many organisms is sequenced, or when trying to distinguish sequencing errors from single nucleotide polymorphisms. While, as discussed in the results section, our model performs well in identifying sequencing errors (our method identifies at least 96.64% of errors in the example PhiX data set), we note that our model may not account for some errors that arise before the sequences enter a flowcell, e.g. during reverse transcription or library amplification. These errors may lack a highly abundant parent sequence and thus are difficult to identify without a reference genome.

A possible direction to improve this model is to include the investigation of the role of single and multiple preceding or following bases in determining error rates. The inclusion in the model of error prone positions, such as those reported in [6,17] is an area of future interest. Correcting for local variants in error rates within lanes, possibly produced by bubbles in flowcells, also warrants further investigation. Additionally, we note that the characteristic phasing-related rise is not visible for all error types in the first 24 bases of GA data. If one were to model the error rate beyond this point one would have to incorporate a second, increasing, exponential in the fitting function or use a more flexible method, such as fitting splines.

## Competing interests

The authors declare that they have no competing interests.

## Authors’ contributions

JS performed the analyses, participated in the design and drafted the manuscript. AS conceived of the study, participated in the design and analyses and helped to draft the manuscript. UB participated in the design and analyses and helped to draft the manuscript. All authors read and approved the final manuscript.

## Supplementary Material

Additional file 1 **Connected subgraph of sequences.** A connected subgraph of sequences of length 21 from an Illumina HiSeq data set. The most abundant sequence in this subgraph occurred 45,484 times and is represented by the largest node (filled circle). Click here for file

Additional file 2 **A larger connected subgraph.** A connected subgraph of sequences of length 21 from an Illumina GA data set. The most abundant sequence in this subgraph occurred 165,504 times in the data set. The size of the nodes (filled circles) representing each sequence is proportional to their abundance. The edges connect sequences that vary in one position only. Click here for file

Additional file 3 **Modelled error rates.** Modelled error rates from a data set with simulated errors according to the pattern found in the data set of Figure 3(a). Click here for file
